# The Effects of Short-Term N-Acetylcysteine Supplementation on Biochemical Parameters in Endurance-Trained Adults: A Randomized Clinical Trial

**DOI:** 10.3390/metabo16070505

**Published:** 2026-07-18

**Authors:** Marcin Sadowski, Emilia Zawieja, Agata Muzsik-Kazimierska, Ewa Bulczak, Agata Chmurzynska

**Affiliations:** Department of Human Nutrition and Dietetics, Poznań University of Life Sciences, Wojska Polskiego 31, 60-624 Poznań, Poland; marcin.sadowski@up.poznan.pl (M.S.); emilia.zawieja@up.poznan.pl (E.Z.); agata.muzsik@up.poznan.pl (A.M.-K.); ewa.bulczak@up.poznan.pl (E.B.)

**Keywords:** thiol supplementation, one-carbon metabolism, antioxidant metabolism, redox homeostasis, sex differences

## Abstract

**Background**: The main aim of this study was to assess the effects of short-term N-acetylcysteine (NAC) supplementation on concentrations of homocysteine (Hcy) and reduced glutathione (rGSH), blood lipid profile and liver enzyme activities in endurance-trained adults, and to determine whether these effects are modified by methylenetetrahydrofolate reductase (*MTHFR*) C677T and glutathione S-transferase Pi 1 (*GSTP1*) A313G. **Methods**: A total of 56 males and 21 females completed a randomized, double-blind, placebo-controlled crossover trial. Participants received 1200 mg of NAC or a placebo for seven days in a crossover design. Serum Hcy and plasma rGSH concentrations were assessed using dedicated biochemical assays, while blood lipid profile and liver enzyme activities were measured using the biochemical analyzer Konelab 20i. Genotyping was conducted using TaqMan probes. A series of within-subject/between-subject repeated-measures analysis of variance (ANOVA) within a general linear model framework were performed to compare Hcy, rGSH, blood lipid profile and liver enzymes activities before and after the intervention. **Results**: Hcy concentrations significantly decreased following NAC supplementation (18.58 ± 5.45 µmol/L vs. 16.51 ± 4.97 µmol/L; *p* = 0.009), although subgroup analysis indicated that the decrease was significant only among females (15.40 ± 4.96 µmol/L vs. 13.60 ± 3.68 µmol/L; *p* = 0.002) without any significant effect among males. We did not observe any significant changes in rGSH, lipid profile, or liver enzyme activities. There was no interaction between NAC supplementation, *MTHFR* and *GSTP1* genotypes and the changes noted in the parameters we analyzed. **Conclusions**: In conclusion, short-term NAC supplementation may reduce circulating Hcy concentrations in endurance-trained adults, particularly in females. No consistent effects were observed for rGSH, lipid profile, or liver enzyme activities.

## 1. Introduction

N-acetylcysteine (NAC) is a synthetic compound that serves as a donor of cysteine, which is a substrate of glutathione (GSH) synthesis. GSH is a major nonenzymatic antioxidant that neutralizes reactive oxygen species (ROS). An active form of GSH, reduced GSH (rGSH) provides electrons to the reaction catalyzed by glutathione peroxidase, which leads to the reduction in ROS and the formation of glutathione disulfide (GSSG) [1]. Intracellular synthesis of rGSH can be limited by cysteine availability, which depends on protein intake and on a transsulfuration pathway [2,3]. For this reason, increased cysteine intake could potentially boost rGSH synthesis. However, cysteine is too unstable to be employed as a supplement [4] and NAC appears to be a better substrate for rGSH synthesis.

The metabolism of rGSH is directly connected with homocysteine (Hcy) metabolism through the transsulfuration pathway [5]. Hcy is a sulfur-containing amino acid whose elevated plasma concentrations are considered a risk factor of cardiovascular and neurodegenerative diseases. In an adult population, normal serum/plasma Hcy concentrations range from 5 to 15 µmol/L and may differ between males and females [6]. Within the transsulfuration pathway, Hcy is metabolized to cystathionine, which is subsequently converted to cysteine, serving as a substrate for rGSH synthesis [5]. Under conditions of oxidative stress and hyperhomocysteinemia, conversion of Hcy to cystathionine may be upregulated, potentially increasing cysteine availability [5]. Deficiencies in the transsulfuration pathway lead to excessive Hcy production and reduced GSH synthesis [7].

NAC supplementation may affect redox balance and rGSH and Hcy metabolism through two main mechanisms: Firstly, by increasing cysteine availability, NAC can directly support rGSH synthesis [8]. Secondly, by modulating Hcy metabolism, NAC can affect the transsulfuration pathway and the cellular homeostasis of sulfur groups [9]. NAC supplementation in an animal model resulted in a significant decrease in Hcy and an increase in cysteine and total GSH concentrations [10]. In humans, long-term NAC supplementation has been associated with improvements in Hcy concentrations, oxidative stress levels, and cardiovascular disease risk [11,12,13,14,15]. Moreover, meta-analyses by Hallajzadeh et al. (2020) and Faghfouri et al. (2020) [11,12] showed that NAC supplementation can significantly reduce Hcy concentrations across diverse populations (healthy individuals, lead-exposed workers, and hemodialyzed older adults) [11,12]. These effects of NAC can be explained by displacing Hcy from protein-binding sites, facilitating the formation of disulfide forms, and increasing liver and kidney clearance [11]. These findings suggest that NAC may modulate redox status via both cysteine–GSH-dependent and Hcy-mediated pathways.

In the general population metabolism is determined as a complex trait, which means that it depends on genetic and environmental factors and interactions between them. As mentioned earlier, Hcy and rGSH concentrations are interrelated and regulated by genetic and lifestyle factors, mainly those associated with folate/methionine metabolism. Polymorphism of the methylenetetrahydrofolate reductase (*MTHFR*) gene is one of the main genetic determinants of this cycle. Specifically, the C677T (rs180133) polymorphism leads to approximately 60% decreased MTHFR enzyme activity in TT homozygotes and 30% in CT heterozygotes, thereby increasing Hcy concentrations, which may further decrease rGSH concentrations [16,17,18]. For this reason, *MTHFR* polymorphism should be considered as a potential factor modifying rGSH concentrations. rGSH activity is also highly dependent on GSTs, enzymes that conjugate rGSH to xenobiotics and peroxides. *GSTP1* A313G (rs1695) is a functional genetic polymorphism with two alleles encoding a protein differing in amino acid sequence (isoleucine/valine) and thus in enzyme activity [19]. Individuals carrying the A allele exhibit approximately 2.6 times lower GSTP1 enzyme activity [20]. Consequently, these polymorphisms may affect detoxification capacity and redox balance. However, its relationship with circulating rGSH concentration has not been well analyzed, which may be particularly relevant under the conditions of dynamic changes in oxidative stress during exercise.

Redox homeostasis and Hcy metabolism play an important role in regulating the response to exercise. Overproduction of ROS may lead to fatigue and delayed recovery; at the same time, moderate ROS production is necessary for training adaptation [21]. Importantly, metabolic changes caused by exercise exceed redox changes and involve increased turnover of molecules dependent on methionine metabolism [22]. This places Hcy in a central role, as a link between the methionine and transsulfuration pathways, making it an indicator of metabolic changes after exercise [22]. NAC may therefore affect the response to exercise not only through its classical role in increasing rGSH synthesis, but also by modulating Hcy concentration within the methionine–transsulfuration pathways. By increasing cysteine availability, NAC can affect GSH synthesis and Hcy, thereby linking redox regulation with methylation-related metabolic demand during repeated exercise bouts. Our previous meta-analysis confirmed that NAC supplementation may be worth considering in order to improve rGSH synthesis in active people, simultaneously reducing IL-6 concentration and muscle soreness [23]. To test the effects of NAC we conducted a study involving trained endurance adults, where we did not observe any significant differences in the results of the sixty-minute time trial between placebo and NAC supplementation [24].

However, whether NAC can affect Hcy and rGSH concentrations in trained adults remains unclear and requires further investigation. This question is particularly important for endurance-trained people who often use NAC before competitions or during intensive training mesocycles, in accordance with the recommendations of the Australian Institute of Sport. Because of the significant impact of exercise on redox balance and Hcy metabolism, the lack of studies that incorporate these factors represents a significant limitation in the literature. Despite growing evidence on the role of NAC in rGSH metabolism, several gaps in the literature remain to be addressed. The majority of studies have examined the effects of NAC supplementation in populations with clinical or metabolic risk, and results from studies with healthy, physically active participants are lacking. Moreover, the effects of NAC on metabolic outcomes were primarily assessed during long-term supplementation protocols, and its short-term effects are not well studied, especially among trained adults. Additionally, because rGSH and Hcy metabolism are affected by genetic variation, functional polymorphism of *MTHFR* C677T and *GSTP1* A313G may be important modifiers of the response to NAC supplementation. Furthermore, sexual dimorphism in GSH metabolism and GSH-dependent response has been reported. Sex-specific differences have been observed in GSH concentrations, the GSH:GSSG ratio, and glutathione transferases (GST) activities, with these parameters being usually higher in females than in males [25]. Similarly, there are sex differences in Hcy metabolism [6], and this aspect also remain insufficiently investigated in the NAC supplementation studies.

Taking the above into consideration, we hypothesized that short-term NAC supplementation would reduce rGSH and Hcy concentrations in healthy, physically active males and females. Therefore, the primary outcomes aim of this study were the concentrations of rGSH and Hcy, whereas the secondary outcomes included basic biochemical parameters such as blood lipids, glucose and liver enzymes activities. We also tested whether NAC exerts sex-specific effects and whether these effects can be modified by *MTHFR* C677T and *GSTP1* A313G polymorphisms. Moreover, some studies have suggested relations between rGSH and Hcy concentrations and body composition outcomes. For example, ref. [26] showed that GSH concentrations are inversely correlated with percentage of body fat (FM%), while ref. [27] observed a positive correlation between Hcy concentration and FM% in healthy, active males. For this reason, we also tested these correlations.

## 2. Materials and Methods

### 2.1. The Study Design

The study was conducted in a double-blind, randomized, placebo-controlled crossover manner. A total of 77 participants received both a NAC and a placebo (PLA) in a randomized order. Each supplementation phase (NAC and PLA) lasted seven days and was separated by a three-week washout period. Participants visited a research laboratory five times during the six-week period. All meetings were conducted in the Department of Human Nutrition and Dietetics, Poznań University of Life Sciences, Poland. Participants were made familiar with the study procedures seven days prior to the start of the study. During meetings 2 to 5, after twelve hours of fasting, blood samples were collected, and body composition was assessed. Recruitment was performed during a period from June 2022 to December 2024. Participants were instructed to maintain their usual diet and not to change their lifestyle or supplementation habits during the study. They were also asked to refrain from taking any antioxidant supplements during the intervention. All subjects were required to give their written informed consent before the commencement of the study, once the experimental procedures, associated risks, and potential benefits of participation had been explained. Participants were randomized using https://www.studyrandomizer.com/ (accessed on 18 October 2024). The randomization process and preparation of the supplements were conducted by a third party not engaged in the experiment; these were revealed only after the study had ended, in order to ensure concealment of the allocation sequence.

Compliance with the protocol was controlled during each meeting. Participants were instructed to bring empty packs after each intervention period. During every meeting participants were asked about consumption of NAC/PLA, consumption of other supplements and the number of workouts during the preceding week.

The study was approved by the local ethical committee (Bioethics Committee, Poznań University of Medical Sciences, Poznań, Poland: decision no. 937/21, 9 December 2021). All procedures were conducted in accordance with the ethical standards of the 1964 Helsinki Declaration. The trial was registered on clinicaltrials.gov (NCT05604586) before the study began.

### 2.2. Participants

One hundred and eight participants were initially enrolled to participate in this study. A total of 77 completed the entire study protocol and were included in the analysis. The criteria for study eligibility included good health, age between 18 and 50 years and at least one year of experience in endurance disciplines and at least three endurance sessions per week during the last year. According to McKay et al. [28] the participants were in the endurance-trained/developmental training status category. The exclusion criteria were current injury or a serious injury within the six months prior to the study, vitamin B or folic acid supplementation during the four weeks preceding the study, and metabolic diseases or other chronic diseases. The exclusion criteria also included significant dietary restrictions among participants, such as a ketogenic or vegan diet. The sample size calculation was performed using G*Power 3.1.9.7. It was estimated that a total of 72 participants was necessary to achieve an anticipated effect size of 0.30 and a power of (1 − ß) = 0.95 at α = 0.05.

### 2.3. Supplementation

Participants were randomly allocated to the PLA-NAC or NAC-PLA groups. The NAC was administered as methylcellulose capsules (Nutropharma, Lesznowola, Poland); each capsule contained 400 mg of NAC. Each participant received instructions to consume three capsules per day with each of the three main meals for a total daily dose of NAC equal to 1200 mg. Capsules were ingested with at least 250 mL of water. Both PLA and NAC capsules looked the same. The order of NAC and PLA supplementation was randomized and double-blinded for both the researchers and participants. The allocation sequence was prepared by a person who was not involved in the study and who had no contact with the participants. Participants were instructed to report any adverse effects during the supplementation period. The allocation sequence was unblinded after the end of the entire investigation.

We applied the commonly accepted protocol. A 7-day supplementation period was selected based on recommendations of the Australian Institute of Sport, which indicate a 4-day supplementation period may be sufficient before competition. Moreover, 7 days of supplementation were sufficient to increase GSH concentrations after exercise [29,30]. Regarding the dose, 1200 mg of NAC is the most frequently chosen dose in the recent research [31].

### 2.4. Body Composition

Body composition was measured in the morning, after a twelve-hour fast, using air-displacement plethysmography with a Bod Pod (Cosmed, Rome, Italy). Body composition analysis followed the manufacturer’s guidelines and standard recommendations for air displacement plethysmography [32]. Fat mass (FM) and fat-free mass (FFM) were calculated using the Siri equation. Thoracic lung volume was estimated using Bod Pod software ver. 5.3.2. During measurement, participants wore only swimsuits and swimcaps.

### 2.5. Blood Collection

Blood samples for DNA isolation and biochemical analyses were collected from the antecubital vein into EDTA and serum-separating tubes. After centrifugation at 1000RCF for 10 min at 4 °C for plasma and 1000RCF for 10 min at 21 °C for serum, samples were stored at −80 °C until biochemical analyses.

### 2.6. Hcy Concentrations Analysis

The serum Hcy concentrations were determined using a fluorometric-based commercially available kit (Abcam, Waltham, USA). The assay principle was based on the reduction in Hcy disulfides to free Hcy, which is broken down by luciferase enzyme. For each test sample, a parallel sample well served as the sample background control. The fluorescence of all samples, background, and standard curve wells was measured at λ_ex_ = 658 nm/λ_em_ = 708 nm in endpoint mode using a microplate reader (Infinite Pro 200, Tecan, Grödig, Austria). The results were calculated as described in the assay instructions.

### 2.7. rGSH Concentrations Analysis

The rGSH kits were purchased from Elabscience (China). The detection working solution and standards were prepared, and standard curves were constructed. Samples and standards were transferred to 96-well plates, where sample absorbance was measured with a microplate reader (Infinite Pro 200, Tecan, Austria), and reduced GSH concentration was quantified in plasma by referencing a standard curve.

### 2.8. Biochemical Analysis

The concentration of total cholesterol (TC), low-density lipoprotein cholesterol (LDL-C), high-density lipoprotein cholesterol (HDL-C), triacylglycerols (TG), glucose and activity of aspartate aminotransferase (AST), alanine aminotransferase (ALT) and gamma-glutamyl transpeptidase (GGTP) were determined using an automated analyzer system (Konelab20i biochemical analyzer, Thermo Electron, Vantaa, Finland).

### 2.9. Dietary Data

For dietary monitoring, participants completed a three-day food diary during NAC and PLA supplementation periods. Dietary intake of energy, macronutrients, micronutrients, and amino acids involved in homocysteine and GSH metabolism (folate, vitamins B6 and B12, glycine, glutamyl acid, cystine, methionine) was assessed. Participants received detailed advice on recording the types of food and drink, the timing of food intake, culinary techniques, and recipes (to be recorded using household measures). Participants were asked to maintain consistent dietary habits throughout the study and to avoid introducing any new supplements. Food diaries were then analyzed for nutrient intake using the Dieta 6.0 software (National Institute of Public Health, National Research Institute, Warsaw, Poland).

### 2.10. DNA Isolation

DNA was isolated from whole blood seven days after blood collection, using a standard kit (NucleoSpinBlood, Macherey-Nagel, Düren, Germany). DNA samples were stored at 4 °C until genotyping was performed.

### 2.11. Genotyping

Genotyping for *MTHFR* C677T (rs180113) and *GSTP1* A313G (rs1695) was performed using TaqMan probes (single-tube assays; Thermo Scientific, Waltham, USA, assay ID C___1202883_20 and C___3237198_20) on a LightCycler480 instrument (Roche Diagnostics, Rotkreuz, Switzerland).

### 2.12. Statistical Analysis

Data normality was assessed using the Shapiro–Wilk test. A series of within-subject/between-subject repeated-measures analysis of variance (ANOVA) within a general linear model framework was used to compare measurements of Hcy, rGSH, TC, HDL-C, LDL-C, TG, glucose, ALT, AST, GGTP concentrations and body composition. The within-subject factors were treatment (NAC and PLA) and time (before and after supplementation). The between-subject factors were *MTHFR* genotype (T-allele carriers vs. CC homozygotes; a dominant model) and *GSTP1* genotype (G-allele carriers vs. AA homozygotes; a dominant model). The sphericity of all variables was examined with Mauchly’s test, and the Greenhouse–Geisser correction was applied when the assumption of sphericity was violated. Post hoc analysis was performed using the Bonferroni test. Differences in dietary intake variables between the NAC and PLA phases were assessed using paired *t*-tests. Correlations between variables were assessed and interpreted using Pearson correlation model. All analysis was performed using Statistica version 14.0.4 (StatSoft, Krakow, Poland). The significance level was set a priori at *p* < 0.05. Effect sizes (partial η^2^) were calculated and interpreted using Cohen’s guidelines. Individual differences in the effect of NAC supplementation were analyzed by calculating the difference between NAC_post_ and NAC_pre_ results. Participants were divided into groups based on changes in rGSH concentrations after NAC supplementation: decreased (nonresponders) and increased (responders). Differences in *MTHFR* and *GSTP1* genotype distributions across the analyzed subgroups were assessed using the Chi-squared test. Differences between nonresponders and responders in rGSH concentration at each time point of intervention were analyzed using the *t*-test for independent variables.

## 3. Results

### 3.1. Participants

**Figure 1:**
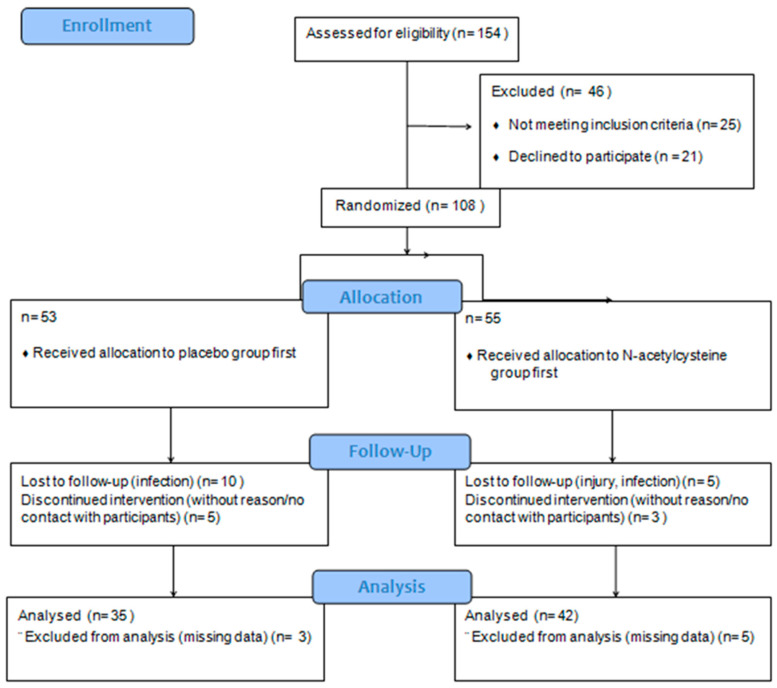
Consort flow diagram of recruited, enrolled, randomized, and analyzed participants.

Out of 108 initially recruited participants, 77 were included in the final analysis, of whom 56 were male and 21 female (Figure 1). At baseline, no significant differences were observed in body mass, FM, or FFM, either across the entire sample or within males and females separately (Table 1). All participants returned their empty supplement packages after the intervention period, so the compliance was 100%. Moreover, no participant reported significant changes in their supplement intakes and the training protocols during the study.

### 3.2. Dietary Intake

There were no statistically significant differences in daily energy or macronutrient intakes between the PLA and NAC supplementation phases (*p* > 0.05 for all comparisons). Mean energy, protein, fat, and carbohydrate intake remained stable across conditions. Folate intake was significantly lower in NAC period of intervention (Supplementary Table S1).

### 3.3. Biochemical Analysis

**Figure 2:**
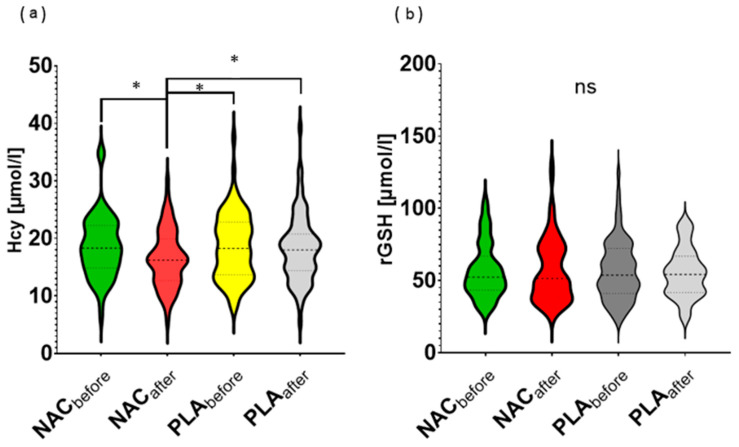
(**a**): Effects of NAC and PLA supplementation on Hcy concentrations in all participants. (**b**): Effects of NAC and PLA supplementation on rGSH concentrations in all participants. * indicates *p* < 0.05.

**Figure 3:**
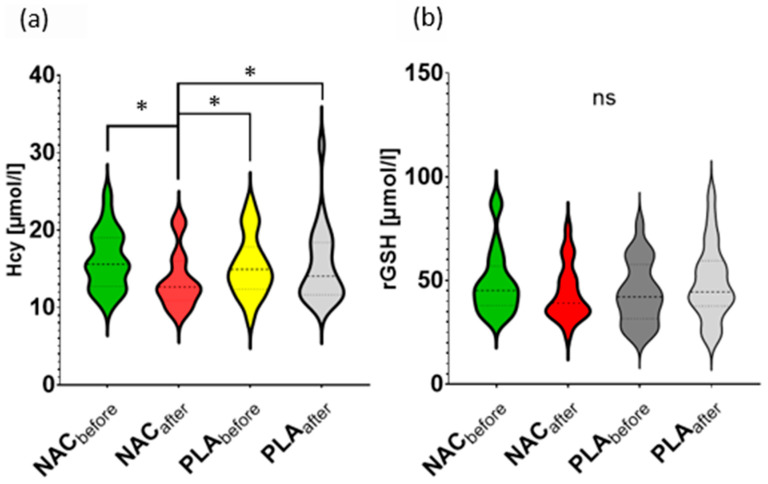
(**a**): Effects of NAC and PLA supplementation on Hcy concentrations in female participants. (**b**): Effects of NAC and PLA supplementation on rGSH concentrations in female participants. * indicates *p* < 0.05.

**Figure 4:**
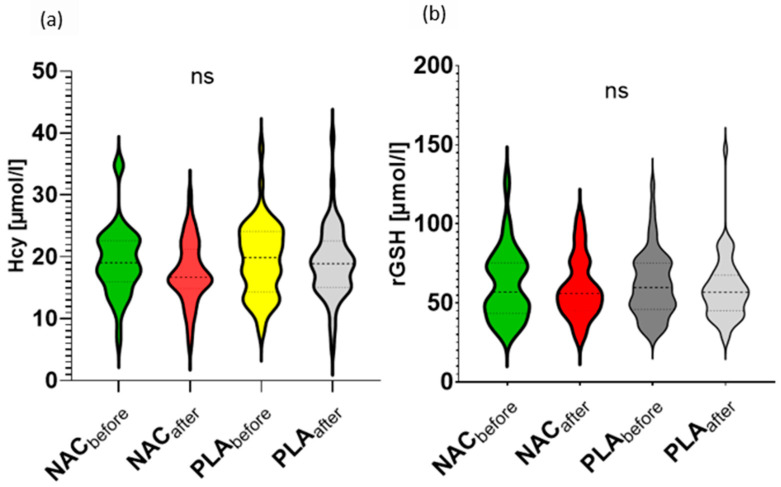
(**a**): Effects of NAC and PLA supplementation on Hcy concentrations in male participants. (**b**): Effects of NAC and PLA supplementation on rGSH concentrations in male participants.

Mean Hcy concentrations in all participants were 18.58 µmol/L prior to PLA supplementation and 18.59 µmol/L prior to NAC supplementation. In males, the corresponding values were 19.80 and 19.51 µmol/L, whereas in females they were 15.35 and 16.11 µmol/L, respectively. Hcy concentrations decreased significantly after NAC supplementation in all participants (Figure 2). However, when we considered sex in the analysis, the differences were significant only among female participants (Figure 3). We did not observe any significant changes in rGSH after NAC supplementation, either in the whole group or in the sex subgroups (Figure 2, Figure 3 and Figure 4).

Individual differences analysis showed that rGSH decreased after NAC supplementation in 34 out of 77 participants. These were further referred to as nonresponders. A general linear model with repeated measures showed that rGSH concentration decreased significantly after NAC supplementation in the nonresponders (*p* = 0.006, Supplementary Table S2) and increased significantly in the responders group (*p* = 0.001) (Supplementary Table S2). Moreover, Hcy concentration also decreased significantly only in the responders group (*p* = 0.005) (Supplementary Table S2).

We did not observe any significant changes in TC, HDL-C, LDL-C, TG, GLU, ALT, and GGTP after NAC supplementation (Table 2). We observed a significant time x treatment interaction for AST activity. However, post hoc analyses did not show any pairwise differences. Analysis of these parameters in the subgroups of male and female participants also did not show any significant differences (Supplementary Table S3).

### 3.4. The Effects of NAC on Hcy and rGSH Concentrations Depend on MTHFR and GSTP1 Genotypes

We also did not observe any effect of *MTHFR* and *GSTP1* polymorphisms on the effectiveness of NAC supplementation on Hcy concentrations (Table 3). Analysis of the *MTHFR* genotype distribution showed a tendency for the CT/TT genotypes to be more frequent among responders than among nonresponders (*p* = 0.059) (Supplementary Table S4).

Similarly, the changes in rGSH after NAC supplementation were not significantly different between *MTHFR* and *GSTP1* genotype groups (Table 4).

### 3.5. Anthropometric Parameters

Baseline body weight (BW) was 80.54 ± 9.47 kg in males and 61.53 ± 6.95 kg in females. The %FM 18.87 ± 6.17% in males and 24.69% ± 6.71% in females while %FFM reached 81.06% ± 6.19% and 75.31% ± 6.72%, respectively. FM was 15.30%Z ± 5.43 kg in males and 15.38 ± 5.34 kg in females while FFM amounted to 65.24 ± 8.30 kg and 46.15 ± 5.02 kg, respectively. We did not observe significant changes in BW, %FM, FM, %FFM or FFM during the intervention in either sex. Concentrations of rGSH were negatively correlated with %FM and FM and positively correlated with %FFM, but only in male participants (Table 5). No correlations were observed between Hcy and body composition outcomes (Table 5).

## 4. Discussion

The main aim of this research was to evaluate the effect of seven-day NAC supplementation on Hcy and rGSH concentrations in healthy trained endurance adults. Moreover, because of sex-determined and genetically determined differences in GSH and Hcy concentrations, we analyzed differences in NAC supplementation effectiveness between males and females, as well as between *MTHFR* and *GSTP1* genotype groups.

In our study, NAC supplementation significantly reduced Hcy concentration. However, subgroup analysis showed that this reduction was significant only among females. Our result showed a decrease in Hcy level by 11.3% (−2.1 µmol/L) in all participants, 9.7% (−1.9 µmol/L) in males, and 15.6% (−2.51 µmol/L) in females. To date, the sex-specific effects of NAC on Hcy have not been reported; however, these results should be interpreted with caution and verified in future research. In male Wistar rats with mild hyperhomocysteinemia, six weeks of NAC supplementation at a dose of 1 g/kg b.w. significantly reduced serum Hcy concentrations [33]. Similarly, Ovrebo et al. (2000) observed decreased concentration of Hcy in rodents after NAC supplementation [10]. Moreover, Wiklund et al. (1996) showed that two-week NAC supplementation at a dose of 2 g per day in a group of eleven patients with Hcy concentration within the normal range resulted in an almost 50% decrease in Hcy concentration [13]. However, in 34 hemodialyzed patients, both males and females, four weeks of NAC supplementation did not significantly decrease Hcy concentration, and the reduction in Hcy was similar to that observed in our group (−2.1 µmol/L) [34].

The reduction in Hcy by NAC can be dose-dependent, which was also shown in the study conducted by Hultberg et al. (1994) [35]. The proposed mechanism of NAC’s effect on Hcy concentration involves NAC-mediated thiol–disulfide exchange, in which NAC displaces Hcy bound to albumin, forming low-molecular-weight disulfides, possibly enhancing its renal clearance [36]. Our results showed that NAC can reduce Hcy concentration in healthy, active people with mild hyperhomocysteinemia, which may be beneficial as the meta-analysis of Wang et al. showed that a 5 µmol/L increase in Hcy concentration is associated with a 22% higher risk of coronary heart disease [37].

In our previous meta-analysis, we reported higher rGSH concentrations after exercise in the NAC-supplemented group compared with the placebo group [23]. Unexpectedly in this study, the rGSH concentration did not change after NAC supplementation. However, subgroup analysis showed that rGSH concentrations increased significantly in the responders, whereas they decreased in the nonresponders following NAC supplementation. A significant reduction in Hcy concentrations was observed only in the responders. Importantly, the rGSH concentration after NAC supplementation did not differ significantly between groups (*p* = 0.062). However, rGSH concentration was significantly lower in the responders than in the nonresponders group before NAC supplementation (*p* < 0.0001). The reduction in Hcy has previously been explained by NAC’s direct effect on Hcy concentration. To our knowledge, the effects of NAC on rGSH and Hcy concentrations have not been examined in the context of individual differences in response to supplementation and this warrants further investigation. Moreover, we are the first to report that Hcy concentrations decreased significantly after NAC supplementation only in participants with increased rGSH concentrations. However, we are aware that exploratory character of this analysis requires validation. As mentioned earlier, rGSH synthesis depends on the availability of cysteine, glycine, and glutamate. As we did not assess cysteine, glycine and glutamine availability in serum, we cannot determine the reason for these differences.

We also did not observe any significant differences in the concentrations of most biochemical outcomes after NAC supplementation. A significant time × treatment interaction in AST concentration was observed. However, the Bonferroni test did not confirm these differences. Contrary to our findings, supplementation with NAC in patients with metabolic syndrome increased HDL-C and decreased TG levels after six weeks of intervention [38]. Moreover, in ovariectomized rats, NAC supplementation decreased serum TC concentration without altering HDL-C concentration [39]. However, Sinaeinejad et al. (2025) showed that eight-week NAC supplementation did not significantly alter concentration of TC, HDL-C, LDL-C, TG, AST, and ALT in patients with metabolic dysfunction-associated steatotic liver disease [14].

Results from our group of participants did not show any interaction between NAC supplementation, *MTHFR*, or *GSTP1* polymorphism on rGSH and Hcy concentrations. We analyzed the distribution of the *MTHFR* genotypes and we found a nonsignificant difference, namely the CT/TT genotypes tended to be more frequent among responders than among nonresponders. The lack of a significant genotype effect may be due to the limited statistical power available to analyze the interaction between genes and nutrients. Moreover, redox homeostasis and one-carbon metabolism depend on multiple factors, including genetic, nutritional, and environmental factors. Our results showed that research with large cohorts is necessary to assess potential interaction between NAC supplementation, changes in rGSH concentration and *MTHFR* gene C677T polymorphism.

As mentioned before, Bordoni et al. (2022) observed a significant negative correlation between GSH and FM% [26]. In our group of participants, we observed a negative correlation between rGSH and FM% and FMkg, but only in males. We also observed a positive correlation in males between rGSH and FFM%, although FFMkg was not significantly correlated with rGSH. Decreased availability of GSH and rGSH in people with higher FM% levels is supported by evidence of ROS overproduction in adipose tissue, and this mechanism is often linked to obesity [26]. Moreover, total cysteine (tCys) levels are associated with BMI and Hcy concentration [40]. A strong linear association between tCys and FM has been reported by Elshorbagy et al. (2008) and this association was not affected by Hcy concentration [41]. In our study, we observed a negative correlation between rGSH and FM% and FMkg. A potential explanation for this result is increased rGSH synthesis, leading to a decline in tCys availability. Lower tCys availability is associated with lower BMI and lower subcutaneous fat levels in patients with homocystinuria [42,43]. However, we did not measure tCys concentration, so we are unable to confirm this hypothesis. In contrast to findings of Zawieja et al. (2022), we did not observe any correlations between Hcy and body composition outcomes [27]. However, the literature about associations between Hcy and body composition outcomes is inconsistent [41]. Elshorbagy et al. (2008) [41] showed that Hcy was negatively associated with FM after adjusting for tCys concentration and were consistent with data from the NHANES study [40,44]. On the other hand, Poirier et al. (2001) reported a positive association between Hcy, body weight and BMI [45]. Observed differences may be associated with variations in population characteristics, methodological differences in body composition assessment but also with adjustment for tCys in the statistical analysis.

Our study has some limitations; we did not measure blood concentrations of GSSG, vitamin B12, vitamin B6, tCys and folate. Vitamins B12 and B6 play an important role in Hcy and GSH metabolism and can affect results, while tCys can be an important factor in analysis of the body composition results. The statistical analysis of genotype groups includes a small number of participants, reducing its power. Moreover, we did not use NAC doses adjusted for weight. We did not control for participants’ training volume or training intensity during the intervention period. We also did not perform carry-over statistical analysis for crossover design and multiple comparison analysis. We acknowledge that failing to perform multiple-comparison analysis could increase the risk of type I error. However, the main outcome of our study was the effect of NAC supplementation on Hcy and rGSH concentrations and remaining aspects of the study were rather exploratory in nature. Moreover, the exploratory character of the responders/nonresponders analysis needs validation. Therefore, these findings should be interpreted with caution.

## 5. Conclusions

In conclusion, our results suggest that short-term NAC supplementation can decrease Hcy concentration in endurance-trained adults. However, this effect may depend on sex and individual metabolic characteristics. Moreover, this supplementation does not affect lipid profile, glucose concentrations or liver enzyme activities. Polymorphism of *MTHFR* (C677T) and *GSTP1* (A313G) do not seem to significantly modify the effect of NAC on Hcy or rGSH, but this should be interpreted with caution, as the sample size was rather small for genetic associations studies.

## Figures and Tables

**Table 1:** Baseline characteristics of participants.

	All (n = 77)	Males (n = 56)	Females (n = 21)
Age (years)	36.6 ± 8.0	37.3 ± 7.6	34.9 ± 9.2
Height (cm)	177.7 ± 9.0	181.3 ± 7.7	168.4 ± 5.0
Body mass (kg)	75.2 ± 12.1	79.1 ± 12.7	60.7 ± 6.6
Training experience (years)	7.0 ± 5.3	7.2 ± 5.4	6.5 ± 5.1
Training units per week	4.7 ± 1.9	4.7 ± 2.0	4.4 ± 1.5

**Table 2:** The effect of NAC and PLA supplementation on lipid profile and glucose concentration and liver enzyme activity.

	NAC_before_ (Mean ± SD)	NAC_after_ (Mean ± SD)	PLA_before_ (Mean ± SD)	PLA_after_ (Mean ± SD)	Time × Treatment
TC [mg/dL]	202.77 ± 35.54	198.02 ± 39.23	202.17 ± 35.68	198.72 ± 38.13	*p* = 0.618 η^2^ = 0.003
HDL-C [mg/dL]	66.43 ± 18.37	66.30 ± 16.93	67.75 ± 16.89	66.17 ± 16.21	*p* = 0.249 η^2^ = 0.019
LDL-C [mg/dL]	113.20 ± 31.11	110.37 ± 32.90	112.06 ± 29.60	110.68 ± 31.36	*p* = 0.259 η^2^ = 0.018
TG [mg/dL]	90.25 ± 44.53	85.81 ± 38.05	86.09 ± 41.99	85.78 ± 38.71	*p* = 0.247 η^2^ = 0.019
GLU [mg/dL]	86.43 ± 9.70	85.21 ± 9.15	86.32 ± 9.13	85.02 ± 9.30	*p* = 0.909 η^2^ = 0.000
AST [U/L]	30.25 ± 13.45	28.71 ± 13.95	29.41 ± 15.63	28.46 ± 10.33	*p* = 0.026 η^2^ = 0.073
ALT [U/L]	22.74 ± 13.00	19.45 ± 9.22	20.75 ± 9.66	20.41 ± 9.11	*p* = 0.259 η^2^ = 0.018
GGTP [U/L]	19.71 ± 9.10	19.01 ± 8.05	20.06 ± 7.43	19.53 ± 7.33	*p* = 0.747 η^2^ = 0.002

PLA_before_: results before PLA supplementation; PLA_after_: results after PLA supplementation; NAC_before_: results before NAC supplementation; NAC_after_: results after NAC supplementation; SD: standard deviation; TC: total cholesterol; HDL: high-density lipoprotein; LDL: low-density lipoprotein; TG: triglycerides; GLU: glucose; AST: aspartate aminotransferase; ALT: alanine aminotransferase; GGTP: gamma-glutamyl transpeptidase. The data were analyzed using a general linear model with repeated measures. The within-subject factors were time (before and after) and treatment (PLA vs. NAC).

**Table 3:** The effect of NAC and PLA supplementation on Hcy concentrations depending on the *MTHFR* and *GSTP1* genotype.

Hcy [µmol/L]						
Group	Genotype	NAC_before_ (Mean ± SD)	NAC_after_ (Mean ± SD)	PLA_before_ (Mean ± SD)	PLA_after_ (Mean ± SD)	
All (n = 77)	*MTHFR* CC (n = 36)	18.50 ± 6.54	16.59 ± 5.67	18.00 ± 6.42	18.06 ± 6.87	Time × Treatment × *MTHFR* *p* = 0.546 η^2^ = 0.005
	*MTHFR* CT/TT (n = 41)	18.65 ± 4.35	16.44 ± 4.33	19.11 ± 5.11	18.45 ± 4.84	
	*GSTP1* AA (n = 38)	18.49 ± 4.61	16.53 ± 4.62	18.45 ± 5.38	18.61 ± 6.28	Time × Treatment × *GSTP1* *p* = 0.974 η^2^ = 0.000
	*GSTP1* AG/GG (n = 39)	18.67 ± 6.22	16.37 ± 5.34	18.72 ± 6.15	17.93 ± 5.44	
Male (n = 56)	*MTHFR* CC (n = 24)	18.55 ± 6.57	16.82 ± 5.76	18.95 ± 6.97	18.41 ± 7.21	Time × Treatment × *MTHFR* *p* = 0.921 η^2^ = 0.000
	*MTHFR* CT/TT (n = 32)	20.00 ± 4.85	17.97 ± 4.43	19.91 ± 4.88	19.52 ± 4.42	
	*GSTP1* AA (n = 26)	19.30 ± 5.17	17.27 ± 4.70	19.09 ± 5.89	19.23 ± 6.77	Time × Treatment × *GSTP1* *p* = 0.334 η^2^ = 0.018
	*GSTP1* AG/GG (n = 30)	19.45 ± 6.11	17.66 ± 5.37	19.84 ± 5.85	18.88 ± 4.81	
Female (n = 21)	*MTHFR* CC (n = 12)	16.97 ± 3.98	14.81 ± 4.43	15.75 ± 4.19	16.49 ± 5.88	Time × Treatment × *MTHFR* *p* = 0.410 η^2^ = 0.040
	*MTHFR* CT/TT (n = 9)	14.98 ± 3.50	11.99 ± 1.32	14.81 ± 3.74	13.96 ± 3.15	
	*GSTP1* AA (n = 12)	16.77 ± 3.30	14.22 ± 3.92	15.81 ± 3.77	15.52 ± 3.89	Time × Treatment × *GSTP1* *p* = 0.490 η^2^ = 0.029
	*GSTP1* AG/GG (n = 9)	15.24 ± 4.49	12.78 ± 3.36	14.73 ± 4.29	15.25 ± 6.39	

PLA_before_: results before PLA supplementation; PLA_after_: results after PLA supplementation; NAC_before_: results before NAC supplementation; NAC_after_: results after NAC supplementation; SD: standard deviation; *MTHFR*: methylenetetrahydrofolate reductase; *GSTP1*: glutathione s-transferase 1. The data were analyzed using a general linear model with repeated measures. The within-subject factors were time (before and after) and treatment (PLA vs. NAC). The between-subject factors were *MTHFR* C677T (CC vs. CT/TT) and *GSTP1* A313G (AA vs. AG/GG) genotype.

**Table 4:** The effect of NAC and PLA supplementation on rGSH concentrations depending on the *MTHFR* and *GSTP1* genotype.

rGSH [µmol/L]						
Group	Genotype	NAC_before_ (Mean ± SD)	NAC_after_ (Mean ± SD)	PLA_before_ (Mean ± SD)	PLA_after_ (Mean ± SD)	
All (n = 77)	*MTHFR* CC (n = 36)	58.18 ± 20.31	55.33 ± 17.24	55.02 ± 20.75	59.84 ± 30.61	Time × Treatment × *MTHFR p* = 0.328 η^2^ = 0.014
	*MTHFR* CT/TT (n = 41)	55.64 ± 23.37	58.00 ± 20.23	58.51 ± 20.66	57.54 ± 22.24	
	*GSTP1* AA (n = 38)	56.45 ± 22.38	57.09 ± 21.74	57.43 ± 21.52	56.12 ± 19.37	Time × Treatment × *GSTP1* *p* = 0.361 η^2^ = 0.012
	*GSTP1* AG/GG (n = 39)	57.19 ± 21.68	56.42 ± 15.73	56.34 ± 20.01	61.05 ± 31.76	
Male (n = 56)	*MTHFR* CC (n = 24)	63.23 ± 21.08	57.57 ± 15.71	58.86 ± 22.29	64.46 ± 33.97	Time × Treatment × *MTHFR p* = 0.683 η^2^ = 0.003
	*MTHFR* CT/TT (n = 32)	60.55 ± 23.90	61.18 ± 21.44	63.68 ± 19.22	66.57 ± 46.46	
	*GSTP1* AA (n = 26)	60.89 ± 23.85	59.55 ± 22.55	62.44 ± 21.52	59.82 ± 17.20	Time × Treatment × *GSTP1* *p* = 0.279 η^2^ = 0.022
	*GSTP1* AG/GG (n = 30)	62.39 ± 21.79	59.69 ± 15.98	60.86 ± 19.95	70.73 ± 53.97	
Female (n = 21)	*MTHFR* CC (n = 12)	48.08 ± 14.71	50.85 ± 19.91	47.32 ± 15.32	50.62 ± 20.72	Time × Treatment × *MTHFR p* = 0.492 η^2^ = 0.028
	*MTHFR* CT/TT (n = 9)	38.20 ± 8.89	46.69 ± 8.90	40.29 ± 14.97	47.67 ± 14.88	
	*GSTP1* AA (n = 12)	46.83 ± 15.61	51.74 ± 19.73	46.58 ± 17.83	48.10 ± 22.08	Time × Treatment × *GSTP1* *p* = 0.956 η^2^ = 0.000
	*GSTP1* AG/GG (n = 9)	39.86 ± 8.53	45.50 ± 8.68	41.27 ± 11.12	51.03 ± 11.90	

PLA_before_: results before PLA supplementation; PLA_after_: results after PLA supplementation; NAC_before_: results before NAC supplementation; NAC_after_: results after NAC supplementation; SD: standard deviation; *MTHFR*: methylenetetrahydrofolate reductase; *GSTP1*: glutathione s-transferase 1. The data were analyzed using a general linear model with repeated measures. The within-subject factors were time (before and after) and treatment (PLA vs. NAC). The between-subject factors were *MTHFR* C677T (CC vs. CT/TT) and *GSTP1* A313G (AA vs. AG/GG) genotype.

**Table 5:** Correlations between Hcy, rGSH and body composition outcomes.

		Hcy [µmol/L]		rGSH [µmol/L]	
		Pearson R^2^	*p*-Value	Pearson R^2^	*p*-Value
Females	BW kg	0.095	0.681	−0.276	0.226
	FM %	−0.115	0.62	−0.408	0.066
	FFM %	0.115	0.62	0.408	0.066
	FM kg	−0.068	0.77	−0.431	0.051
	FFM kg	0.189	0.412	0.074	0.751
Males	BW kg	−0.259	0.054	−0.072	0.600
	FM %	−0.068	0.618	−0.327	0.014
	FFM %	0.068	0.618	0.327	0.014
	FM [kg]	−0.136	0.317	−0.347	0.009
	FFM [kg]	−0.196	0.147	0.132	0.331

Hcy: homocysteine, rGSH: reduced glutathione; BW: body weight; FM%: fat mass percentage; FFM%: fat-free mass percentage; FM: fat mass; FFM: fat-free mass.

## Data Availability

The data supporting reported results are available on request from the corresponding author (A.C.).

## Supplementary Materials

The following supporting information can be downloaded at https://www.mdpi.com/article/10.3390/metabo16070505/s1: Supplementary Table S1. Dietary intake during PLA and NAC supplementation periods; Supplementary Table S2: Effects of NAC supplementation on rGSH and Hcy concentration in responder and nonresponder groups Supplementary Table S3: Effects of NAC and PLA supplementation on blood lipid profile, glucose concentrations, and liver enzyme activities depending on sex; Supplementary Table S4: Chi-squared analysis of differences in genotype distribution in the group with increased and decreased rGSH after NAC supplementation. Supplementary Material File S1: CONSORT checklist.

## Abbreviations

The following abbreviations are used in this manuscript:

NAC	N-acetylcysteine
GSH	Glutathione
rGSH	Reduced glutathione
Hcy	Homocysteine
*MTHFR*	Methylenetetrahydrofolate reductase
*GSTs*	Glutathione transferases
*BW*	Body weight
FM	Fat mass
FM%	Fat mass percentage
FFM	Fat-free mass
FFM%	Fat-free mass percentage
TC	Total cholesterol
LDL-C	Low density lipoprotein
HDL-C	High density lipoprotein
TG	Triglycerides
GLU	Glucose
ALT	Alanine aminotransferase
AST	Aspartate aminotransferase
GGTP	Gamma-glutamyl transferase
tCys	Total cysteine
