# Supplementary material for: The Effects of Short-Term N-Acetylcysteine Supplementation on Biochemical Parameters in Endurance-Trained Adults: A Randomized Clinical Trial
Source: Metabolites. 2026 Jul 18;16(7):505. doi: 10.3390/metabo16070505 (PMC13414332; doi:10.3390/metabo16070505)
Supplement: Supplementary file 1 [file metabolites-16-00505-s001.zip › metabolites-4383893-supplementary.pdf]

Supplementary Table S1. Dietary intake during PLA and NAC supplementation periods

	NAC	PLA	P
Energy intake [kJ/day]	11 146 ± 3 460	10 652 ± 2 326	0.196
Protein [g/day]	119 ± 33	122 ± 41	0.685
Fat [g/day]	84 ± 26	90 ± 37	0.127
Carbohydrates [g/day]	331 ± 84	347 ± 121	0.259
Glutamic acid [g/day]	23.6 ± 6.9	23.8 ± 8.0	0.768
Cystine [mg/day]	1828.09 ± 543.5	1872.3 ± 621.2	0.528
Methionine [mg/day]	2830.8 ± 857.7	2880.0 ± 1087.1	0.676
Glycine [mg/day]	4589.4 ± 1417.6	4546.3 ± 1574.09	0.830
Folate [µg/day]	403.3 ± 144.2	480.3 ± 301.6	0.045
Vitamin B12 [µg/day]	4.9 ± 2.8	5.8 ± 3.9	0.131
Vitamin B6 [mg/day]	2.55 ± 1.0	2.63 ± 0.9	0.586

PLA: values from the PLA supplementation period; NAC: values from the NAC supplementation period; SD: standard deviation.

The data were analyzed using a *t*-test for the dependent variable.

Supplementary Table S2. Effects of NAC supplementation on rGSH and Hcy concentration in responder and nonresponder groups

		NAC_{before} (mean±SD)	NAC_{after} (mean±SD)	PLA_{before} (mean±SD)	PLA_{after} (mean±SD)	Time x treatment
rGSH (μmol/L)	Nonresponders (n = 34)	68.45 ± 22.28b	52.26 ± 18.51a	60.58 ± 20.50bc	59.52 ± 22.97ac	$p = 0.006$ $\eta^2 = 0.300$
	Responders (n =43)	47.64 ± 16.76a	60.30 ± 18.49b	53.95 ± 20.52	53.90 ± 16.92	$p = 0.001$ $\eta^2 = 0.223$
Hcy (μmol/L)	Nonresponders (n = 34)	19.01 ± 6.11	17.65 ± 5.65	19.34 ± 6.31	18.20 ± 6.02	$p = 0.748$ $\eta^2 = 0.003$
	Responders (n =43)	18.25 ± 4.91	15.61 ± 4.21a	18.00 ± 5.26	18.31 ± 5.77	$p = 0.005$ $\eta^2 = 0.169$

rGSH: reduced glutathione; Hcy: homocysteine, SD: standard deviation;

The data underwent general linear model analysis with repeated measures. The within-subject factor included time (pre and post) and treatment (PLA x NAC).

Supplementary Table S3. Effects of NAC and PLA supplementation on blood lipid profile, glucose concentrations, and liver enzyme activities depending on sex;

	Sex	NAC$_{before}$ (mean±SD)	NAC$_{after}$ (mean±SD)	PLA$_{before}$ (mean±SD)	PLA$_{after}$ (mean±SD)	Time x Treatment
CHOL [mg/dl]	Female (n = 21)	209.10 ± 31.06	203.73 ±28.14	206.07 ± 27.07	203.37 ± 28.81	p = 0.599 η^2= 0.017
	Male (n = 56)	200.41 ± 37.06	195.87 ± 42.68	200.71 ± 38.53	196.97 ± 41.19	p = 0.980 η^2= 0.000
HDL-C [mg/dl]	Female (n = 21)	76.00 ± 20.00	75.42 ± 16.01	77.32 ± 15.03	74.30 ± 13.44	p = 0.584 η^2= 0.018
	Male (n = 56)	62.84 ± 16.52	62.87 ± 16.10	64.16 ± 16.24	63.13 ± 16.21	p = 0.293 η^2= 0.023
LDL-C [mg/dl]	Female (n = 21)	113.05 ± 21.64	108.99 ± 22.07	110.20 ± 21.42	110.86 ± 21.45	p = 0.170 η^2= 0.108
	Male (n = 56)	113.25 ± 34.17	110.89 ± 36.30	112.76 ± 32.28	110.62 ± 35.52	p = 0.875 η^2= 0.001
TG [mg/dl] ALT [U/L]	Female (n = 21)	72.52 ± 24.34	65.43 ± 22.07	64.33 ± 21.25	68.49 ± 25.31	p = 0.199 η^2= 0.095
	Male (n = 56)	97.02 ± 46.64	93.46 ± 40.14	94.25 ± 21.25	92.27 ± 40.99	p = 0.551 η^2= 0.007
GLU [mg/dl]	Female (n = 21)	83.72 ± 6.08	83.02 ± 4.52	84.83 ± 5.51	83.27 ± 7.01	p = 0.641 η^2= 0.013
	Male (n = 56)	87.45 ± 10.62	86.03 ± 10.28	86.88 ± 10.15	85.67 ± 10.01	p = 0.855 η^2= 0.001
AST [U/L]	Female (n = 21)	29.92 ± 13.22	24.97 ± 5.88	24.78 ± 7.27	25.91 ± 7.72	p = 0.087 η^2= 0.162
	Male (n = 56)	28.69 ± 10.02	27.24 ± 9.47	28.44 ± 9.17	28.66 ± 9.53	p = 0.100 η^2= 0.055
ALT [U/L]	Female (n = 21)	19.97 ± 8.45	17.14 ± 5.74	17.61 ± 6.79	17.01 ± 4.37	p = 0.226 η^2= 0.085
	Male (n = 56)	23.78 ± 14.27	20.32 ± 10.12	21.92 ± 10.34	21.68 ± 10.08	p = 0.279 η^2= 0.022
GGTP [U/L]	Female (n = 21)	17.24 ± 11.01	16.07 ± 8.15	16.98 ± 6.98	15.53 ± 6.42	p = 0.948 η^2= 0.000

	Male (n = 56)	20.63 ± 8.19	20.12 ± 7.80	21.21 ± 7.32	21.02 ± 7.14	$p = 0.564$ $\eta^2 = 0.006$

PLA_{before}: results before PLA supplementation; PLA_{after}: results after PLA supplementation; NAC_{before}: results before NAC supplementation; NAC_{after}: results after NAC supplementation; SD: standard deviation; CHOL: total cholesterol; HDL: high density lipoprotein, LDL: low density lipoprotein; TG: triglycerides; GLU: glucose; AST: aspartate aminotransferase; ALT: alanine aminotransferase; GGTP: gamma glutamyl- transpeptidase;

The data underwent general linear model analysis with repeated measures. The within-subject factor included time (pre and post) and treatment (PLA x NAC).

Supplementary Table S4. Chi-squared analysis of differences in genotype distribution in the group with increased and decreased rGSH after NAC supplementation

	Genotype	Responders group	Nonresponders group	Chi-Square (χ^2)	p -value
rGSH	*MTHFR* CT/TT	27	14	3.58	0.059
	MTHFR CC	16	20		
	GSTP1 AG/GG	21	18	0.13	0.720
	GSTP1 AA	22	16		

rGSH: reduced glutathione; *MTHFR*: methylenetetrahydrofolate reductase; *GSTP1*: glutathione s-transferase 1.
